# Patient-reported negative interpersonal experiences in Polish primary care and their association with healthcare avoidance: a large-scale cross-sectional study

**DOI:** 10.1186/s12913-026-15097-7

**Published:** 2026-07-08

**Authors:** Agnieszka J. Szczepek, Malgorzata Wrzosek, Marta Makowska

**Affiliations:** 1 Department of Otorhinolaryngology, Head and Neck Surgery, Charité- Universitätsmedizin Berlin, Corporate Member of Freie Universität Berlin and Humboldt-Universität zu Berlin, 10117 Berlin, Germany; 2 Faculty of Medicine and Health Sciences, University of Zielona Góra, Zielona Góra, 65- 046 Poland; 3 Faculty of Psychology and Cognitive Science, Adam Mickiewicz University, Poznań, 60-568 Poland; 4 Department of Economic Psychology, Kozminski University, Warszawa, 03-301 Poland

**Keywords:** Primary healthcare, Patient-reported experiences, Healthcare avoidance, Medical humanities, Trust in physicians

## Abstract

**Background:**

Negative interpersonal experiences in primary healthcare may undermine trust in physicians and continuity of care, yet population-level evidence linking such experiences to healthcare avoidance remains limited. This study, therefore, quantified the prevalence of such experiences in Polish primary care and examined their association with appointment cancellations.

**Methods:**

A large-scale, cross-sectional, quota-based online survey was conducted among 1,075 adults in Poland. Negative healthcare experiences and reasons for canceling medical appointments were summarized using two composite indices: the Index of Negative Healthcare Experiences (INHE; 0–14) and the Index of Reasons for Canceling Medical Appointments (IRCMA; 0–11). Associations with sociodemographic factors, trust in physicians, and satisfaction with care were analyzed.

**Results:**

The most frequently reported negative experiences were a lack of empathy (43.6%), long waiting times that adversely affected health (38.0%), and feeling ignored (37.6%). The mean INHE score was 4.2 ± 4.3 (median 3.0). Higher INHE scores were associated with lower trust in physicians (*r* = -.20) and lower satisfaction with current primary care physician services (*r* = -.22; both *p* < 0.001). Chronic disease (β = 0.235) and lower life satisfaction (β = -0.167) were the strongest predictors of negative experiences. The most commonly reported reasons for appointment cancellation were prior negative experiences with a physician (38.1%) and physician rudeness (36.3%).

**Conclusions:**

In this large online sample, negative interpersonal experiences in primary care were common and were associated with lower trust in physicians and self-reported appointment cancellation.

**Supplementary Information:**

The online version contains supplementary material available at https://doi.org/10.1186/s12913-026-15097-7.

## Introduction

Primary care physicians represent the most frequent point of contact between the healthcare system and the general population. Beyond clinical competence, the quality of these encounters depends strongly on humanistic skills, including effective communication, empathy, respect for dignity, and the ability to manage emotionally demanding situations [1]. In response to these needs, medical education in many countries has increasingly incorporated courses in the medical humanities – drawing on psychology, sociology, ethics, and related disciplines – to foster patient-centered care and strengthen professional–patient relationships [2–5]. In Poland, the medical humanities are a compulsory component of medical curricula [6].

While these educational efforts emphasize the importance of relational competencies, their relevance is especially evident in everyday primary care practice. Well-functioning primary healthcare plays a critical role in disease prevention, timely diagnosis, continuity of care, and overall patient well-being [7]. When patients disengage from primary care by changing physicians or canceling scheduled appointments, these processes may be disrupted, potentially resulting in delayed treatment, poorer health outcomes, and increased inefficiency in the system. Moreover, as artificial intelligence (AI) increasingly provides health advice, it could decrease certain primary care visits, create new alternatives to traditional doctor-led care, impact how patients decide when to seek medical help, and alter the doctor–patient relationship—especially in terms of communication and empathy [8]. Understanding the factors associated with patients’ decisions to discontinue or avoid medical consultations is therefore of considerable clinical, ethical, and public health relevance.

To date, research has identified a broad range of factors that are relevant to patients’ decisions to change physicians. A recent systematic review by Ribeiro et al. identified seven thematic categories explaining why patients change physicians [9]. These include the doctor–patient relationship, consultation convenience, referrals and recommendations, medication and treatment concerns, practice management and costs, personal preferences and physician attributes, and accessibility. Among these, two domains are particularly closely related to the humanization of medicine: Theme 1 (doctor–patient relationship) and Theme 6 (personal preferences and physician attributes). These themes encompass interpersonal and relational aspects of care, such as empathy, communication quality, perceived respect, trust, and attitudes toward physicians’ personal characteristics.

Despite this conceptual framework, significant gaps remain in the empirical literature. Although previous research has examined patient satisfaction and healthcare utilization, empirical evidence remains limited regarding the cumulative burden of negative interpersonal experiences in primary care and their contribution to healthcare-avoidance behaviors, including appointment cancellations [9]. As a result, the mechanisms linking everyday relational failures in primary care with patients’ decisions to disengage from care remain insufficiently understood.

These gaps are especially noticeable when examined from an ethical perspective. Negative interpersonal experiences in healthcare cannot be reduced to individual dissatisfaction; they may reflect broader patterns of inequity and discrimination. Patients might choose to cancel medical appointments based on physicians’ personal traits, such as preferring a doctor of the same sex, which some may believe offers increased safety, comfort, and better communication. At the same time, repeated experiences of disrespect, dignity violations, or being ignored by primary healthcare workers may disproportionately affect socially or medically vulnerable groups, including those with lower economic status. These phenomena challenge core ethical principles of medical practice, including respect for persons, justice, and nonmaleficence, and underscore the moral responsibility of healthcare systems and professionals to ensure equitable, dignity-based care.

Against this background, the present study addresses these gaps by systematically examining negative healthcare experiences in Polish primary care and analyzing how such experiences are associated with patients’ decisions to cancel medical consultations. Using a large, quota-based online survey of adults in Poland, the study aims to (1) quantify the prevalence of adverse interpersonal experiences in primary healthcare, (2) identify sociodemographic factors associated with their occurrence, and (3) assess how negative experiences and physician-related attributes relate to healthcare avoidance, trust in physicians, and satisfaction with care. By focusing on the humanistic and ethical dimensions of everyday medical encounters, this study contributes to a more comprehensive understanding of how relational quality in primary care shapes patient behavior and continuity of care.

## Methods

### Study design and sample

The study employed a large, quota-based, cross-sectional online survey conducted in Poland between July 16 and 23, 2025, using the Ariadna research panel. A quota-based sampling strategy was used to approximate the demographic structure of the adult Polish population, based on data from the Central Statistical Office. Quotas were established based on sex (two categories), age (five categories), and residential size (five categories), creating a total of 50 strata. For education level and province, marginal distributions were constrained to match the population proportions, without accounting for interactions with other quota variables.

Because a substantial proportion of the Polish population does not use the internet (approximately 23%) [10], the sample cannot be considered fully representative of the general population. Nevertheless, this approach enabled efficient data collection in Poland.

## Sample size calculation

The sample size was calculated to estimate the prevalence of negative interpersonal experiences in primary care at the 95% confidence level, with a margin of error of ± 3%. Sample size estimation was based on the standard formula for estimating a single-population proportion (*n = Z*^*2*^*p(1 - p)/d*^*2*^) [11]. A conservative expected prevalence of 50% was assumed, as the true population prevalence was unknown. With a 95% confidence level (*Z* = 1.96) and a margin of error of ± 3% (*d* = 0.03), the minimum required sample size was 1,067 participants. The final analytical sample comprised 1,075 respondents, thereby satisfying the predefined precision criterion.

## Eligibility and exclusion criteria

Respondents were eligible to participate if they were adults aged 18 years or older, resided in Poland, were registered members of the Ariadna online research panel, were able to complete the questionnaire in Polish, and provided electronic informed consent.

Respondents were excluded from the analytical dataset if they declined to participate or did not meet the eligibility criteria. A total of 1,820 respondents began the survey. Of these, 745 were excluded before the final analytical dataset was established. The largest group of exclusions comprised respondents who did not meet the study criteria, declined participation, or provided inconsistent demographic information, including a mismatch between the declared age category and year of birth. Because these reasons were recorded together, they are reported as a combined category (*n* = 558). In addition, 136 respondents did not complete the questionnaire. A further 51 responses were excluded during data quality control because the survey completion time was more than three standard deviations above or below the mean, suggesting potentially invalid or inattentive responses. The mean survey completion time was 21 min and 50 s. After all exclusions, the final analytical sample comprised 1,075 respondents.

## Survey administration and incentives

Participants were recruited through a commercial online research panel that uses a points-based incentive system. Respondents who did not meet eligibility criteria received one panel point as standard compensation for their time spent in the screening stage. In contrast, respondents who completed the survey in accordance with the study requirements received twenty points as the standard panel incentive. Panel points are a form of non-monetary incentive that can be accumulated and exchanged by panel members for various rewards (e.g., books, cosmetics) available on the panel provider’s platform.

## Survey instrument and pilot testing

The complete survey comprised 19 substantive questions and 15 sociodemographic items. The questionnaire was pilot-tested with 13 participants before fielding, and their feedback was incorporated into the final version; revisions included simplifying question wording, clarifying ambiguous items, and refining the instructions.

The present analysis focused on four main questionnaire components: two multi-item sets assessing negative healthcare experiences and reasons for canceling or refusing medical appointments, and two single-item measures assessing trust in physicians and satisfaction with current primary care physician services. Additional physician-related preference items concerning sex, age, and nationality were used descriptively where relevant. The sociodemographic and health-related variables included in the analyses were sex, age group, place of residence, education level, self-rated health, financial situation, life satisfaction, chronic disease status, and household composition. Sex was recorded as a binary categorical variable, age was assessed in five predefined categories, and place of residence was classified into five categories reflecting settlement size. Education was measured using ordered categories reflecting increasing educational attainment. Self-rated health and financial situation were recoded into three categories: good or very good, moderate, and bad or very bad. Life satisfaction was categorized as satisfied or rather satisfied, dissatisfied or rather dissatisfied, difficult to say, and missing/other. Chronic disease status was coded as a binary variable indicating whether the respondent reported any chronic disease requiring ongoing medical care. Household composition was assessed by counting the number of adults and children in the respondent’s household. For regression analyses, dichotomous variables were entered as binary indicators, and ordered categorical variables were coded ordinally where appropriate. The section of the questionnaire used in this study, along with the anonymized dataset, is publicly accessible at https://doi.org/10.6084/m9.figshare.30948947.

### Ethical considerations

The study was conducted in accordance with the Declaration of Helsinki and was approved by the Research Ethics Committee of Kozminski University, Warsaw, Poland, decision no. 07/07/2025. All participants received information about the study’s purpose, the voluntary nature of participation, data anonymity, and the option to discontinue participation before submitting the questionnaire. Electronic informed consent was required before access to the questionnaire was granted. No personally identifying data were included in the analytical dataset.

## Measures

### Negative healthcare experiences

Negative experiences in healthcare were assessed using 14 dichotomous items (yes/no) describing adverse interpersonal and organizational situations encountered during contact with primary healthcare services. These items included, for example, a lack of empathy, insufficient consultation time, communication problems, violations of dignity and privacy, inappropriate treatment, and perceived disrespect. The selection of items was informed by prior literature on patient–physician communication, healthcare experiences, patient satisfaction, and healthcare avoidance, supporting the face and content validity of the item set. Because the INHE was developed for this study and has not been externally validated, it should be interpreted as an exploratory cumulative index rather than as a fully validated psychometric scale.

An Index of Negative Healthcare Experiences (INHE) was constructed by summing affirmative responses across the 14 items, yielding a score range from 0 to 14. The index demonstrated excellent internal consistency with a Cronbach’s α of 0.915, supporting the use of the summed score as an exploratory cumulative index. The INHE was the primary outcome variable in this study and, in regression analyses, served as the dependent variable.

### Reasons for canceling medical appointments

Reasons for canceling or refusing medical appointments were assessed using 11 dichotomous items that reflected both experiential factors (e.g., prior negative interactions, rudeness, poor communication) and physician-related characteristics (e.g., sex, age, nationality, appearance, sexual orientation). The item selection was informed by prior literature on physician switching, appointment avoidance, patient preferences, and physician-related characteristics, supporting the face and content validity of the item set. Because the IRCMA was developed for this study and has not been externally validated, it should be interpreted as an exploratory cumulative index.

An Index of Reasons for Canceling Medical Appointments (IRCMA) was calculated by summing affirmative responses, yielding a score ranging from 0 to 11. The index demonstrated high internal reliability (Cronbach’s α = 0.825).

### Trust in physicians and satisfaction with care

Trust in physicians was assessed using the survey question “In general, do you trust medical doctors?” Responses were recorded on a 10-point slider ranging from 1 (“not at all”) to 10 (“very much”). This method has been used in previous research [12, 13]. Such global measures of trust are commonly operationalized with a single item and have been widely used in large-scale population surveys, including the World Values Survey, to capture overall trust in medical professionals and healthcare institutions [14].

Satisfaction with the currently selected primary healthcare physician was assessed using a single item from the survey question “How satisfied are you with the services provided by your currently selected primary healthcare physician?” Responses were recorded on a 5-point Likert-type scale, ranging from 1 (“definitely no”) to 5 (“definitely yes”). For clarity, this variable is referred to as satisfaction with current primary care physician services. Such a response format is widely used in studies evaluating patient satisfaction with primary care [15].

Both measures were selected for their established use in large-scale surveys and their demonstrated face validity in capturing evaluations. Both variables were assessed with single-item ordinal measures and treated as continuous in correlational analyses. This approach is common in survey-based health research, particularly when Likert-type scales include at least five response categories, which allows them to approximate continuous distributions without substantially biasing parameter estimates [16, 17].

### Statistical analysis

Statistical analyses were conducted using IBM SPSS Statistics, version 29. Associations between categorical variables were examined using chi-square tests of independence. To facilitate more nuanced interpretation, standardized residuals (SR) were analyzed, with values exceeding ± 1.9 indicating statistically meaningful deviations from expected frequencies.

Relationships between continuous variables were assessed using Pearson correlation coefficients, interpreted according to conventional thresholds: 0.1 (weak), 0.3 (moderate), and 0.5 (strong) [18]. Pearson correlation coefficients were used because the INHE and IRCMA were cumulative sum scores with multiple ordered score levels, and trust in physicians was measured on a 10-point scale, thereby allowing these variables to be treated as approximately continuous for estimating linear associations. Satisfaction with care was measured on a 5-point Likert-type scale and treated as approximately continuous, consistent with common practice in large survey studies when Likert-type variables have at least 5 response categories. Given the large analytical sample, estimates of association and corresponding significance tests were considered robust to moderate deviations from normality. Correlation coefficients were interpreted primarily by magnitude and direction rather than by statistical significance alone.

To identify sociodemographic predictors of negative healthcare experiences, multiple linear regression analysis was performed with the INHE score as the dependent variable. Predictors included age (continuous), sex, education level, self-rated health, life satisfaction, presence of chronic disease, financial situation, type of locality, and household composition. Model assumptions were evaluated before analysis. Autocorrelation of residuals was excluded using the Durbin–Watson statistic (1.914). Linearity and homoscedasticity were assessed visually, and no systematic violations were observed. Although normality tests (Kolmogorov–Smirnov and Shapiro–Wilk) were statistically significant, this was not considered problematic given the large sample size and established robustness of linear models under such conditions [19]. Multicollinearity diagnostics (variance inflation factors, eigenvalues, and condition indices) indicated no significant collinearity issues.

The threshold for statistical significance was set at *p* < 0.05 for all analyses.

## Results

### Sample characteristics

The study included 1,075 adults living in Poland, of whom 52.7% were female, with a mean age of 47.6 years (SD = 16.1) and households averaging 2.5 adults (SD = 1.1). Just over a quarter (27.8%) had completed higher education, and just over two-thirds (68.2%) relied exclusively on public health insurance through the National Health Fund (NFZ). Sample characteristics are presented in Table 1.

**Table 1:** Characteristics of the analytical sample (*N* = 1,075)

Characteristic	Category	*n*	%
Sex	Female	566	52.7
	Male	509	47.3
Age group	18–24 years	91	8.5
	25–34 years	182	16.9
	35–44 years	217	20.2
	45–54 years	177	16.5
	55 years or older	408	38.0
Place of residence	Rural area	426	39.6
	Town up to 20,000 inhabitants	139	12.9
	Town 20,000–99,999 inhabitants	208	19.3
	City 100,000-500,000 inhabitants	176	16.4
	City over 500,000 inhabitants	126	11.7
Education	Primary	43	4.0
	Lower secondary	25	2.3
	Basic vocational	305	28.4
	Secondary	403	37.5
	Higher, bachelor/engineer level	55	5.1
	Higher, master level	231	21.5
	Doctoral or higher	13	1.2
Self-rated health	Good or very good	482	44.8
	Moderate	432	40.2
	Bad or very bad	141	13.1
	Missing / other	20	1.9
Financial situation	Good or very good	412	38.3
	Moderate	495	46.0
	Bad or very bad	139	12.9
	Missing / other	29	2.7
Life satisfaction	Satisfied or rather satisfied	707	65.8
	Dissatisfied or rather dissatisfied	197	18.3
	Difficult to say	136	12.7
	Missing / other	35	3.3
Chronic disease	Yes	386	35.9
	No	689	64.1
Number of adults in household	1	172	16.0
	2	496	46.1
	3	215	20.0
	4	131	12.2
	5	37	3.4
	6 or more	24	2.2

### Negative healthcare experiences

Respondents reported a high prevalence of negative experiences in their interactions with healthcare providers (Supplementary Table 1). The most frequently reported experiences were lack of empathy from physicians (43.6%), prolonged waiting times that adversely affected health (38.0%), and feeling ignored or disregarded (37.6%).

**Fig. 1:**
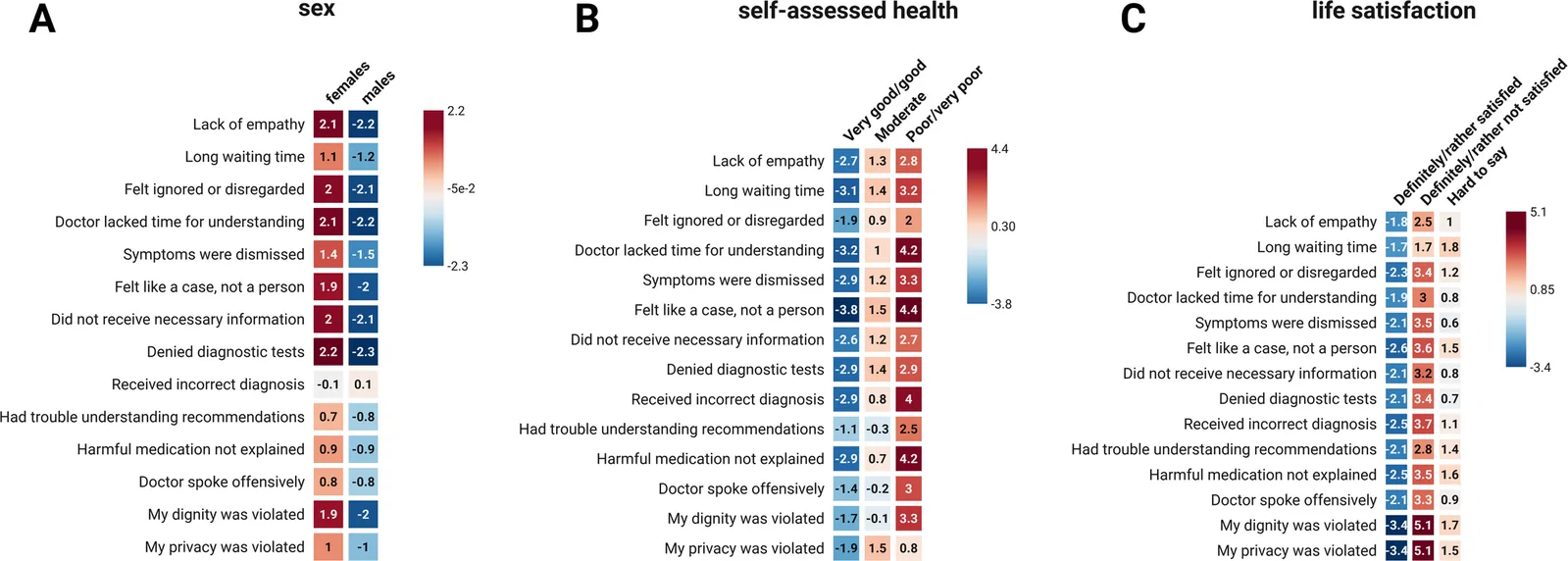
Standardized residual heatmaps showing associations between respondent characteristics and reported negative healthcare experiences. Panels show associations between negative healthcare experiences and (**A**) sex, (**B**) self-assessed health, and (**C**) life satisfaction. Numbers within cells represent standardized residuals from chi-square tests. Positive values indicate that a given respondent group reported a specific negative experience more often than expected under the assumption of independence, whereas negative values indicate less frequent reporting than expected. Residual values exceeding ± 1.9 were interpreted as statistically meaningful deviations from expected frequencies

As shown in Fig. 1, reporting of negative experiences varied systematically across groups. Females, respondents in poorer self-assessed health, and those dissatisfied with life were generally overrepresented among those reporting adverse encounters, whereas respondents in good health or satisfied with life were underrepresented. The largest deviations concerned relational and dignity-related items – lack of empathy and feeling ignored among female respondents, and being treated as a case rather than a person, incorrect diagnosis, unexplained harmful medication, and violations of dignity and privacy among respondents in poorer health or with lower life satisfaction. The complete set of standardized residuals is presented in Fig. 1 (panels A–C).

INHE scores ranged from 0 points (31.6%) to 14 points (4.4%). The mean INHE score was 4.2 ± 4.3, with a median of 3.0 and an interquartile range of 7.0 (Q1 = 0, Q3 = 7). Higher INHE scores were weakly but significantly associated with lower trust in physicians (r(1073) = -0.20, *p* < 0.001) and lower satisfaction with current primary care physician services (r(1038) = -0.22, *p* < 0.001).

To identify sociodemographic factors associated with negative healthcare experiences, a multiple linear regression was performed with INHE as the dependent variable (Table 2). The model was statistically significant (F(9, 1015) = 20.890; *p* < 0.001) and explained 15.6% of the variance in INHE scores (R² = 0.156; adjusted R² = 0.149). Multicollinearity diagnostics indicated no relevant violations of model assumptions, with variance inflation factors ranging from 1.02 to 1.48.

The strongest predictors of higher INHE scores were the presence of chronic disease (β = 0.235; on average 2.117 points higher on the INHE) and lower life satisfaction (β = -0.167). Increasing age, larger households, and good self-rated health were associated with fewer negative experiences, whereas higher education and female sex were associated with more; financial situation and type of locality were not significant predictors in the adjusted model. Full estimates are reported in Table 2.

**Table 2:** Estimates of linear regression parameters for sociodemographic predictors of the INHE index

Variable	B	SE	β	t	*p*
Age (continuous variable)	-0.043	0.009	-0.159	-5.018	< 0.001
Number of adults in the household (continuous variable)	-0.267	0.115	-0.07	-2.317	0.021
Higher education and other (1 = higher)	0.876	0.281	0.091	3.119	0.002
Type of locality (1 = large/much larger)	0.005	0.282	0.001	0.017	0.986
Health (1 = good or very good)	-0.795	0.306	-0.091	-2.595	0.010
Financial situation (1 = good or very good)	-0.371	0.288	-0.042	-1.288	0.198
Life satisfaction (1 = definitely/rather yes)	-1.558	0.305	-0.167	-5.112	< 0.001
Chronic diseases (1 = yes)	2.117	0.279	0.235	7.58	< 0.001
Sex (1 = female)	0.765	0.253	0.088	3.021	0.003
(Constant)	6.542	0.642		10.183	< 0.001

### Reasons for canceling medical appointments

Physicians’ personal and interpersonal characteristics were also reported in relation to respondents’ decisions to cancel or refuse medical appointments. Most respondents reported no preference regarding the physician’s sex (62.9%), while 19.1% indicated that sex preferences depended on the situation. Among respondents for whom physician sex mattered, preferences favored female physicians (13.2%) over male physicians (4.8%). The majority considered physician age (87.9%) and nationality (71.7%) unimportant.

Respondents were further asked whether specific physician-related characteristics had ever led them to cancel or refuse medical care (Supplementary Table 2). The most frequently reported reasons were previous negative experiences with a given physician (38.1%), physician rudeness (36.3%), and negative opinions about the physician, such as online reviews (35.3%).

**Fig. 2:**
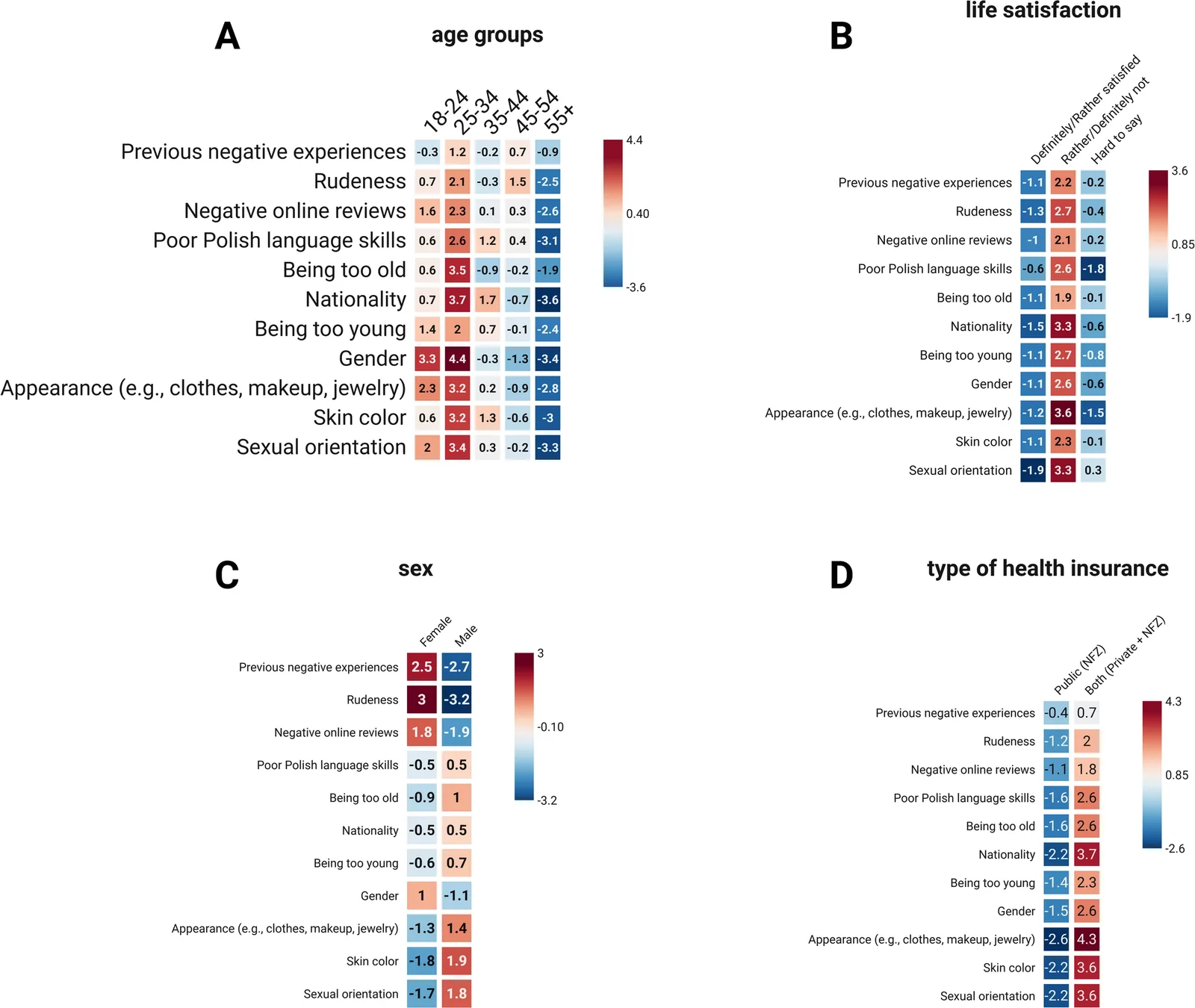
Standardized residual heatmaps showing associations between respondent characteristics and reasons for canceling medical appointments. Panels show associations between reasons for canceling or refusing medical appointments and (**A**) age group, (**B**) life satisfaction, (**C**) sex, and (**D**) type of health insurance. Numbers within cells represent standardized residuals from chi-square tests. Positive values indicate that a given respondent group reported a specific reason more often than expected under the assumption of independence, whereas negative values indicate less frequent reporting than expected. Residual values exceeding ± 1.9 were interpreted as statistically meaningful deviations from expected frequencies

As summarized in Fig. 2, citing physician-related characteristics (e.g., sex, nationality, appearance, skin color, sexual orientation, and poor command of Polish) was concentrated among younger respondents – particularly those aged 25–34 – and among those with private or mixed insurance, whereas respondents dissatisfied with life were overrepresented across multiple cancellation reasons. Sex differences followed a relational pattern: female respondents more often cited prior negative interactions and physician rudeness, whereas male respondents were comparatively more likely to cite the physician’s skin color. The complete standardized residuals are shown in Fig. 2 (panels A–D).

IRCMA scores ranged from 0 points (45.0%) to 11 points (1.6%). The mean IRCMA score was 1.7 ± 2.3, with a median of 1.0 and an interquartile range of 3.0 (Q1 = 0.0; Q3 = 3.0). IRCMA and INHE were moderately positively correlated (r(1073) = 0.41, *p* < 0.001), and IRCMA scores were weakly and negatively associated with trust in physicians (r(1073) = -0.12, *p* < 0.001) and satisfaction with current primary care physician services (r(1038) = -0.14, *p* < 0.001).

## Discussion

For most patients in Poland, the family physician in primary healthcare represents the first point of contact when health concerns arise. At this stage, the diagnostic and therapeutic pathway typically begins; consequently, negative experiences early in care may be associated with subsequent patterns of care. If patients develop reluctance or distrust toward their physician at this initial point, this may be associated with differences in subsequent help-seeking behavior, continuity of care, and treatment outcomes [20]. Prior research has demonstrated that negative healthcare experiences are associated with changing physicians or facilities, reduced healthcare utilization, and withdrawal from further consultations, underscoring their broader clinical and public health significance [9, 21].

The present study demonstrates that negative interpersonal experiences in Polish primary care are both common and cumulative. A substantial proportion of respondents reported multiple adverse encounters, as reflected by the distribution of INHE scores. Importantly, the most frequently reported experiences – lack of empathy, feeling ignored, insufficient consultation time, and poor communication – were predominantly relational rather than technical. These findings are consistent with previous literature showing that interpersonal factors strongly shape patients’ perceptions of care quality [9, 22]. Communication quality has been repeatedly linked to patient understanding, satisfaction, treatment adherence, and trust, and, in some contexts, is perceived by patients as more salient than physicians’ clinical expertise [23].

These findings reinforce the relevance of medical humanities and humanization-of-medicine approaches in everyday clinical practice. Communication and empathy are not innate traits but professional competencies that can be systematically developed. Evidence from meta-analyses indicates that empathy training is effective [24]; however, in Poland, the formal integration of humanistic competencies into medical education standards has occurred only recently. Such competencies were absent from the 2019 standards and were explicitly introduced only in 2023 (https://eli.gov.pl/eli/DU/2023/2152/ogl/pol#). Moreover, the scope, duration, and format of humanization-related teaching vary widely across Polish medical schools [6]. Our results indicate that this variability could have real-world consequences: many current physicians appear insufficiently prepared to manage the relational aspects of modern primary care. This may be associated with more negative patient experiences and lower engagement with healthcare. Therefore, future initiatives to enhance patient-centered care should focus not only on communication skills training in medical education but also on strengthening interpersonal and relational skills during the selection process and ongoing professional development of physicians.

Beyond interpersonal communication, organizational constraints - limited physician availability and long waiting times - were a significant source of dissatisfaction. A large proportion of respondents reported waiting times that adversely affected their health, a finding consistent with national surveys indicating a mismatch between patient expectations and actual access to primary healthcare services. These results align with earlier studies that identify accessibility as a key area for improvement in Polish primary care [25]. Importantly, such challenges reflect systemic pressures rather than individual physician failings, especially in urban areas where workforce shortages and high demand prolong waiting times [26]. Nevertheless, from the patient’s perspective, organizational shortcomings and interpersonal failures are often experienced as a single, cumulative burden.

The associations identified by our regression model should be interpreted with caution because patient-reported healthcare experiences are complex and inherently subjective. Unmeasured factors probably also influenced the observed variability. However, the analysis revealed several variables associated with higher INHE scores, notably having a chronic disease, lower life satisfaction, and younger age, which showed the largest standardized coefficients in the model. Other significant predictors included self-rated health, education level, sex, and the number of adults in the household.

The cumulative nature of negative experiences is particularly evident among patients with chronic diseases, who emerged as the most vulnerable group in our analyses. Chronic illness was the strongest predictor of higher INHE scores. This finding is consistent with evidence from other healthcare systems indicating that patients with chronic conditions – especially multimorbidity – receive poorer care in primary care, particularly regarding access, continuity, and communication [27]. Reduced health-related quality of life, including chronic pain, likely amplifies sensitivity to deficiencies in care. Moreover, patients with chronic conditions interact with the healthcare system more frequently, increasing both their exposure to potential shortcomings and the consequences of relational failures.

Life satisfaction was also independently associated with patient-reported experiences. Individuals with higher life satisfaction reported fewer negative encounters, whereas those dissatisfied with life reported a markedly higher burden of adverse experiences and a greater propensity to cancel appointments. This pattern may reflect differences in cognitive appraisal: individuals with higher well-being may be less likely to interpret ambiguous situations as negative. Prior research suggests that higher life satisfaction is associated with more favorable evaluations of both treatment outcomes and interpersonal aspects of care [28]. At the same time, reduced life satisfaction and well-being are associated with increased interpersonal stress and negative perceptions of social relationships [29], which can also influence how healthcare interactions are perceived.

Older respondents reported fewer negative experiences, consistent with studies showing more positive evaluations of healthcare among older patients [30]. In the Polish context, this may be partly explained by historically shaped, more paternalistic physician–patient relationships, which may be associated with lower expectations regarding communication style, patient autonomy, and shared decision-making.

Although other sociodemographic characteristics showed comparatively smaller effect sizes in our model, previous research suggests that they may still play a meaningful role in shaping patients’ healthcare experiences.

Poorer self-rated health was associated with a greater number of reported negative experiences, consistent with the findings of Schwei et al. [21], who demonstrated that individuals reporting worse health were more likely to report negative healthcare experiences. Previous research has also indicated that patients who rate their health as poorer tend to report worse experiences, such as less prompt access to care and lower patient-centeredness [31]. Comparable results were reported in a Danish study, in which poorer self-rated health was strongly associated with less favorable assessments of general practitioner care [32]. One possible explanation is that individuals with poorer self-rated health tend to use healthcare services more frequently, thereby increasing their exposure to systemic barriers.

Higher educational attainment was also associated with higher INHE scores. This finding aligns with previous literature indicating that individuals with higher levels of education tend to evaluate healthcare experiences more critically [21]. In their systematic review, Crow et al. (2003) found that higher education levels were more often linked to lower reported satisfaction with healthcare, with few studies showing the opposite [33]. This trend might be due to higher expectations among more educated individuals concerning communication, patient autonomy, and patient-centered care.

Females reported negative healthcare experiences more frequently than males. However, the literature on sex differences in negative healthcare experiences remains inconclusive. Some studies have found no significant differences between females and males in reporting adverse healthcare experiences [34]. Conversely, Elliott et al. (2012) reported results consistent with ours, indicating that female patients generally judged their healthcare experiences less favorably, particularly in areas such as medication communication, discharge details, and cleanliness. Their research specifically examined hospital care rather than primary healthcare [35].

Several mechanisms may contribute to these observed sex differences. They may reflect differences in expectations regarding healthcare, variations in how females communicate health concerns, and potential differences in how female patients are perceived and treated within healthcare settings. Previous studies conducted in Poland have demonstrated that females tend to hold higher expectations of primary care physicians than males [36]. Moreover, Weisman (1987) observed that female patients are generally more likely to ask questions and participate actively during medical consultations, which may translate into higher expectations for information exchange, physician engagement, and the relational aspects of care [37]. An additional explanation may be that females, on average, use primary care services more frequently than males due to higher rates of healthcare-seeking behavior [38], thereby increasing cumulative exposure to potentially negative encounters.

Our research indicates that living in households with more adults may be associated with increased social support. This support could help individuals cope with healthcare challenges and lessen the perceived burden of negative experiences.

A key finding is that appointment cancellations co-occurred with a higher cumulative burden of negative healthcare experiences, as reflected in the moderate correlation between INHE and IRCMA. Prior negative interactions, physician rudeness, and unfavorable online opinions were the most common reasons for canceling visits. These findings highlight the role of indirect experiences and digital information environments. While information about physicians was historically shared primarily through personal networks, online reviews and rating platforms now significantly influence patient choices, especially when assessing service quality in advance is challenging [39].

Notably, a subset of respondents reported canceling appointments based on physicians’ personal characteristics, including language proficiency, age, nationality, sex, appearance, skin color, and sexual orientation. These findings raise essential interpretative and ethical questions. On the one hand, such responses cannot be automatically equated with prejudice. Patients may rely on visible or socially salient characteristics as heuristics to reduce uncertainty when evaluating professional competence in advance, which is difficult. On the other hand, some responses may reflect underlying biases or stereotypes. The present study cannot disentangle these mechanisms; motivations may include fear of uncomfortable interactions, lack of trust, prior negative experiences, or preferences related to intimacy and perceived safety.

Stricter expectations regarding physician characteristics were most pronounced among younger respondents and those with private or mixed (private and public) insurance coverage. These groups typically have greater access to alternatives and can more easily disengage from unsatisfactory care. Younger individuals generally enjoy better health [40] and higher digital and eHealth literacy [41], enabling them to seek information and alternative providers more efficiently. Private insurance further expands choice and lowers the threshold for switching providers. In this context, appointment cancellation may function as a rational risk-minimization strategy rather than a sign of disengagement per se. The growing use of digital health resources should be understood as complementing, rather than replacing, interpersonal relationships and communication in clinical care.

Individuals with lower life satisfaction were more likely than those who were more satisfied to cite physicians’ personal characteristics - such as appearance, sexual orientation, or nationality - as reasons for canceling a medical appointment. This pattern may reflect the fact that lower life satisfaction is often associated with greater irritability [42], lower tolerance for difference [43], and reduced social trust [44], which together increase the likelihood of perceiving a medical encounter as uncomfortable or threatening. In this context, appointment cancellation may function as a coping strategy to avoid anticipated discomfort [45] rather than as a response to objective deficiencies in care.

At the same time, clear sex differences were evident. Females were more likely to cancel appointments for communication-related reasons, whereas males more frequently cited physician-related characteristics, including skin color. These findings suggest that cancellations among female respondents are more often associated with their perceptions of interaction quality, consistent with evidence that female patients generally place greater value on physicians’ communication skills and interpersonal style [46].

Taken together, the present study extends existing literature by moving beyond single indicators of dissatisfaction and showing that a higher cumulative burden of negative interpersonal experiences co-occurred with self-reported appointment cancellation. This study highlights how incorporating relational, organizational, and sociodemographic factors, along with explicitly addressing the ethically sensitive aspects of patient decision-making, reinforces the importance of humanistic skills in maintaining trust, continuity, and equity in primary healthcare.

### Limitations and future directions

Several limitations of this study should be acknowledged. First, the study used a quota-based online survey, which limits the generalizability of the findings. Although quotas were designed to reflect the demographic makeup of the Polish adult population, approximately 23% of the population who do not use the internet were inevitably left out. This exclusion likely results in the underrepresentation of older people, individuals with lower socioeconomic status, and those with limited digital literacy, who may exhibit different healthcare usage patterns and have unique experiences with primary care, including potential barriers to access.

Furthermore, participation in a research panel may introduce self-selection bias because panel members are experienced at completing surveys and may differ from the general population in attitudes, expectations, or reporting styles. These differences could affect how negative healthcare experiences and cancellation behaviors are reported, potentially skewing the estimated prevalence. Consequently, the results should be viewed as indicative of patterns within a large online sample rather than fully representative of the entire population. Additionally, all measures relied on self-reports and retrospective recall. Negative healthcare experiences and reasons for canceling appointments were reported subjectively and may have been influenced by recall bias, current emotional state, or general attitudes toward healthcare. The cross-sectional design further precludes causal inference. At the same time, negative experiences were associated with healthcare avoidance, trust issues, and patient satisfaction with care; however, the exact direction of these relationships over time cannot be conclusively determined. Furthermore, although the study captured a wide range of adverse experiences and reasons for appointment cancellation, it did not include direct information about the physicians involved in the reported encounters. Characteristics such as the physician’s actual sex, age, nationality, language proficiency, or professional role were not measured, limiting the ability to determine whether reported reasons for cancellation reflected objective mismatches, prior concrete experiences, or more generalized attitudes and expectations. Similarly, the study cannot disentangle whether cancellations attributed to physician characteristics stemmed from prejudice, fear of uncomfortable interactions, prior negative experiences, or preferences related to perceived safety and intimacy. While the measures captured patients’ overall experience of the healthcare system, they could obscure differences between care settings that have distinct organizational constraints and interactional norms.

In addition, reliance on single-item measures of trust and satisfaction may limit the assessment of reliability and the granularity of these constructs, a limitation that should be considered when interpreting the findings.

Future research should build on these findings in several ways. Longitudinal studies are needed to clarify causal pathways between negative healthcare experiences, declining trust, and healthcare avoidance, including whether repeated adverse encounters lead to progressive disengagement from care. Future surveys should also directly measure physician characteristics associated with negative experiences and cancellations, enabling a more precise examination of the roles of concordance, communication barriers, and perceived differences. Qualitative studies could provide deeper insight into how patients interpret and narrate “negative” encounters, how they weigh interpersonal versus organizational factors, and how they make sense of physician-related characteristics when deciding to attend or cancel appointments. In particular, qualitative approaches may help distinguish heuristic decision-making under uncertainty from explicit prejudice or discrimination. Finally, intervention-oriented research is warranted to test whether strengthening communication skills, empathy, and organizational accessibility in primary care can reduce the cumulative burden of negative experiences and improve continuity of care, especially among vulnerable groups such as patients with chronic diseases or low life satisfaction. Integrating such evidence with medical education and health policy may help translate humanistic competencies from formal curricula into everyday clinical practice.

## Conclusions

In this large online sample, negative interpersonal experiences in Polish primary care were common and varied across sociodemographic and health-related groups. Higher INHE scores were associated with lower trust in physicians and with a greater number of self-reported reasons for canceling or refusing appointments. Appointment cancellations may reflect responses to repeated relational failures during medical interactions. These results underscore the importance of the quality of patient–provider relationships for ongoing care and ethical practice. Enhancing humanistic skills, addressing implicit bias, and creating organizational environments that promote respectful, patient-centered communication are essential to reducing healthcare avoidance and ensuring equitable primary care.

## Supplementary Information

Below is the link to the electronic supplementary material.

Supplementary Material 1

## Data Availability

The anonymized dataset and the complete survey questionnaire are publicly available at (https://figshare.com/articles/dataset/Dataset_on_Patient-Reported_Negative_Interpersonal_Experiences_in_Polish_Primary_Healthcare/30948947).

## References

[1] Teutsch C. Patient-doctor communication. Med Clin North Am. 2003;87(5):1115–45. 10.1016/s0025-7125(03)00066-x. 14621334

[2] Qian Y, Han Q, Yuan W, Fan C. Insights into medical humanities education in China and the West. J Int Med Res. 2018;46(9):3507–17. 10.1177/0300060518790415. PMC6135995 30088424

[3] Carr SE, Noya F, Phillips B, Harris A, Scott K, Hooker C, et al. Health Humanities curriculum and evaluation in health professions education: a scoping review. BMC Med Educ. 2021;21(1):568. 10.1186/s12909-021-03002-1. PMC8579562 34753482

[4] Coronado-Vázquez V, Antón-Rodríguez C, Gómez-Salgado J, Ramírez-Durán MDV, Álvarez-Montero S. Evaluation of learning outcomes of humanities curricula in medical students. A meta-review of narrative and systematic reviews. Front Med (Lausanne). 2023;10:1145889. 10.3389/fmed.2023.1145889. PMC10150636 37138737

[5] Zhang X, Li L, Zhang Q, Le LH, Wu Y. Physician Empathy in Doctor-Patient Communication: A Systematic Review. Health Commun. 2024;39(5):1027–37. 10.1080/10410236.2023.2201735. 37062918

[6] Dec-Pietrowska J, Szczepek AJ. A Study of differences in compulsory courses offering medicine humanization and medical communication in polish medical schools: content analysis of secondary data. Int J Environ Res Public Health. 2021;18(24). 10.3390/ijerph182413326. PMC8706785 34948930

[7] Liss DT, Uchida T, Wilkes CL, Radakrishnan A, Linder JA. General Health Checks in Adult Primary Care: A Review. JAMA. 2021;325(22):2294–306. 10.1001/jama.2021.6524. 34100866

[8] Blease C, Kaptchuk TJ, Bernstein MH, Mandl KD, Halamka JD, DesRoches CM. Artificial Intelligence and the Future of Primary Care: Exploratory Qualitative Study of UK General Practitioners’ Views. J Med Internet Res. 2019;21(3):e12802. 10.2196/12802. PMC6446158 30892270

[9] Ribeiro MC, Martins E, Prazeres F. Why Do primary care patients change their physicians: an overview of the literature. Int J Environ Res Public Health. 2025;22(2). 10.3390/ijerph22020285. PMC11855251 40003510

[10] Korzystanie z internetu. w023 roku [Internet usage in 2023]. Public opinion research center (CBOS); 2023.

[11] Lwanga SK, Lemeshow SWHO. Sample size determination in health studies: a practical manual. 1991.

[12] Gerkens S, Lefèvre M, Bouckaert N, Levy M, Maertens de Noordhout C, Obyn C, et al. Performance of the Belgian health system: Report 2024. KCE Reports. Brussels, Belgium: Belgian Health Care Knowledge Centre (KCE); 2024. p. 168.

[13] European foundation for the improvement of living and working conditions: trust in institutions, EU. 2020–2024 (scale 1–10). 2025. https://www.eurofound.europa.eu/en/surveys-and-data/data-catalogue/trust-institutions-eu-2020-2024-scale-1-10. Accessed 8 May 2026.

[14] Ewald L, Bellettiere J, Farag TH, Lee KM, Palani S, Castro E, et al. Association Between trust in health care professionals and health care access: insights from an online survey across 21 countries. Int J Public Health. 2025;70–2025. 10.3389/ijph.2025.1607884 PMC12018240 40276462

[15] Friedel AL, Siegel S, Kirstein CF, Gerigk M, Bingel U, Diehl A, et al. Measuring patient experience and patient satisfaction—how are we doing it and why does it matter? A comparison of european and U.S. American Approaches. Healthcare. 2023. 10.3390/healthcare11060797. PMC10048416 36981454

[16] Norman G. Likert scales, levels of measurement and the laws of statistics. Adv Health Sci Educ. 2010;15(5):625–32. 10.1007/s10459-010-9222-y. 20146096

[17] Carifio J, Perla R. Resolving the 50-year debate around using and misusing Likert scales. Med Educ. 2008;42(12):1150–2. 10.1111/j.1365-2923.2008.03172.x. 19120943

[18] Cohen J. Statistical power analysis for the behavioral sciences. Routledge. 2013.

[19] Schmidt AF, Finan C. Linear regression and the normality assumption. J Clin Epidemiol. 2018;98:146–51. 10.1016/j.jclinepi.2017.12.006 29258908

[20] Shukla M, Schilt-Solberg M, Gibson-Scipio W. Medical mistrust: a concept analysis. Nurs Rep. 2025;15(3). 10.3390/nursrep15030103. PMC11944586 40137676

[21] Schwei RJ, Johnson TP, Matthews AK, Jacobs EA. Perceptions of negative health-care experiences and self-reported health behavior change in three racial and ethnic groups. Ethn Health. 2017;22(2):156–68. 10.1080/13557858.2016.1244621. PMC5548094 27748134

[22] Buja A, Cavinato M, Perissinotto E, Rausa G, Mastrangelo G, Toffanin R. Why do patients change their general practitioner? Suggestions on corrective actions. Ir J Med Sci. 2011;180(1):149–54. 10.1007/s11845-010-0571-6. 20835893

[23] Street RL, Makoul G, Arora NK, Epstein RM. How does communication heal? Pathways linking clinician–patient communication to health outcomes. Patient Educ Couns. 2009;74(3):295–301. 10.1016/j.pec.2008.11.015. 19150199

[24] Paulus CM, Meinken S. The effectiveness of empathy training in health care: a meta-analysis of training content and methods. Int J Med Educ. 2022;13:1–9. 10.5116/ijme.61d4.4216. PMC8995011 35092671

[25] Krztoń-Królewiecka A, Oleszczyk M, Windak A. Do Polish primary care physicians meet the expectations of their patients? An analysis of Polish QUALICOPC data. BMC Fam Pract. 2020;21(1):118. 10.1186/s12875-020-01190-1. PMC7313208 32576153

[26] OECD, Economic Surveys OECD. Poland 2025. Paris, France: OECD Publishing; 2025.

[27] Paddison CA, Saunders CL, Abel GA, Payne RA, Campbell JL, Roland M. Why do patients with multimorbidity in England report worse experiences in primary care? Evidence from the General Practice Patient Survey. BMJ Open. 2015;5(3):e006172. 10.1136/bmjopen-2014-006172. PMC4386239 25805528

[28] Arboleda AM. Satisfaction with life and perception of healthcare services. Int J Hospitality Manage. 2023;110:103450. 10.1016/j.ijhm.2023.103450.

[29] Moksnes UK, Haugan G. Stressor experience negatively affects life satisfaction in adolescents: the positive role of sense of coherence. Qual Life Res. 2015;24(10):2473–81. 10.1007/s11136-015-0977-8. 25833012

[30] Adams CWR, Schembri A, Sharma A, Walpola R, Harrison R. Does Patient Experience Change with Age? Exploring Associations Between Patient Experience, Gender and Age. Patient Experience J. 2024;11(1):141–8. 10.35680/2372-0247.1890.

[31] Zhong A, Davie S, Wang R, Kiran T. Understanding disparities in primary care patient experience. Can Fam Physician. 2021;67(7):e178–87. 10.46747/cfp.6707e178. PMC8279669 34261726

[32] Heje HN, Vedsted P, Sokolowski I, Olesen F. Patient characteristics associated with differences in patients’ evaluation of their general practitioner. BMC Health Serv Res. 2008;8(1):178. 10.1186/1472-6963-8-178. PMC2533311 18715502

[33] Crow R, Gage H, Hampson S, Hart J, Kimber A, Storey L, Thomas H. The measurement of satisfaction with health care: implications for practice from a systematic review of the literature. Health Technol Assess. 2003;6(32):244. 10.3310/hta6320. 12925269

[34] Petek D, Künzi B, Kersnik J, Szecsenyi J, Wensing M. Patients’ evaluations of European general practice–revisited after 11 years. Int J Qual Health Care. 2011;23(6):621–8. 10.1093/intqhc/mzr052. 21831966

[35] Elliott MN, Lehrman WG, Beckett MK, Goldstein E, Hambarsoomian K, Giordano LA. Gender differences in patients’ perceptions of inpatient care. Health Serv Res. 2012;47(4):1482–501. 10.1111/j.1475-6773.2012.01389.x. PMC3401395 22375827

[36] Szczepek AJ, Wrzosek M, Makowska M. Attributes Desired in a General Practitioner by Adults: Implications for Humanization in Medicine and Enhancement of the Patient Experience. Healthcare. 2024. 10.3390/healthcare12242589. PMC11727714 39766016

[37] Weisman CS. Communication between women and their health care providers: research findings and unanswered questions. Public Health Rep. 1987;102(4 Suppl):147–51. PMC1478023 3120213

[38] Bertakis KD, Azari R, Helms LJ, Callahan EJ, Robbins JA. Gender differences in the utilization of health care services. J Fam Pract. 2000;49(2):147–52. 10718692

[39] Rothenfluh F, Schulz PJ. Physician Rating Websites: What Aspects Are Important to Identify a Good Doctor, and Are Patients Capable of Assessing Them? A Mixed-Methods Approach Including Physicians’ and Health Care Consumers’ Perspectives. J Med Internet Res. 2017;19(5):e127. 10.2196/jmir.6875. PMC5432667 28461285

[40] Wierucki Ł, Kujawska-Danecka H, Mossakowska M, Grodzicki T, Błędowski P, Chudek J, et al. Health status and its socio-economic covariates in the older population in Poland – the assumptions and methods of the nationwide, cross-sectional PolSenior2 survey. Arch Med Sci. 2022;18(1):92–102. 10.5114/aoms.2020.100898. PMC8826695 35154530

[41] Zhang Y, Rosli Y, Mohd Saat NZ, Abd Talib R, Tang S. Scoping review of the e-health literature in older adults. Digit HEALTH. 2025;11:20552076251362545. 10.1177/20552076251362545. PMC12368412 40851928

[42] Gröndal M, Ask K, Winblad S. The distinction between irritability and anger and their associations with impulsivity and subjective wellbeing. Sci Rep. 2023;13(1):10398. 10.1038/s41598-023-37557-4. PMC10300089 37369760

[43] Tuzov V, Boateng RA, Understanding Sexual Prejudice Across European Nations. The Role of Religiosity, Democratic Values, and Happiness. Sexuality Res Social Policy. 2025;22(4):1778–93. 10.1007/s13178-025-01084-w.

[44] Zhang RJ. Social trust and satisfaction with life: A cross-lagged panel analysis based on representative samples from 18 societies. Soc Sci Med. 2020;251:112901. 10.1016/j.socscimed.2020.112901. 32182443

[45] Kite ME, WhitleyJr BE. Sex Differences in Attitudes Toward Homosexual Persons, Behaviors, and Civil Rights A Meta-Analysis. Pers Soc Psychol Bull. 1996;22(4):336–53. 10.1177/0146167296224002.

[46] Schmittdiel J, Grumbach K, Selby JV, Quesenberry CP. Jr. Effect of physician and patient gender concordance on patient satisfaction and preventive care practices. J Gen Intern Med. 2000;15(11):761–9. 10.1046/j.1525-1497.2000.91156.x. PMC1495609 11119167

