# Supplementary material for: Patient-reported negative interpersonal experiences in Polish primary care and their association with healthcare avoidance: a large-scale cross-sectional study
Source: BMC Health Serv Res. 2026 Jul 8;26:1352. doi: 10.1186/s12913-026-15097-7 (PMC13632397; doi:10.1186/s12913-026-15097-7)
Supplement: Supplementary file 1 [file 12913_2026_15097_MOESM1_ESM.docx]

**Supplementary Table 1.** Sex, self-rated health, and life satisfaction versus the experience of hostile situations in healthcare contact.

| **Survey items** | **Total** | **Sex, N=1075** | | | | **Self-assessment of health, N=1055** | | | | | **Satisfaction of life, N=1040** | | | | |
| --- | --- | --- | --- | --- | --- | --- | --- | --- | --- | --- | --- | --- | --- | --- | --- |
|  |  | **Female** | **Male** | **Chi^2^** | **p** | **Very good and good** | **Moderate** | **Very bad and bad** | **Chi^2^** | **p** | **Very good and good** | **Very bad and bad** | **Hard to say** | **Chi^2^** | **p** |
| I encountered a lack of empathy from the doctor or other medical staff | 43.6% (469) | 49.5% (280) | 37.1% (189) | 16.6 | **<.001** | 35.7% (172) | 47.9% (207) | 59.6% (84) | 30.1 | **<.001** | 39.5% (279) | 55.8% (110) | 49.3% (67) | 18.6 | **<.001** |
| I experienced a long wait for an appointment, which negatively affected my health | 38.0% (409) | 41.0% (232) | 34.8% (177) | 4.4 | **.036** | 29.7% (143) | 42.6% (184) | 55.3% (78) | 35.8 | **<.001** | 34.5% (244) | 46.2% (91) | 47.8% (65) | 14.6 | **<.001** |
| I felt ignored or disregarded by the doctor | 37.6% (404) | 42.8% (242) | 31.8% (162) | 13.6 | **<.001** | 32.4% (156) | 40.5% (175) | 48.2% (68) | 13.9 | **<.001** | 32.5% (230) | 52.8% (104) | 44.1% (60) | 29.5 | **<.001** |
| The doctor did not have enough time to fully understand my health problem | 36.5% (392) | 41.7% (236) | 30.6% (156) | 14.1 | **<.001** | 27.8% (134) | 39.6% (171) | 58.2% (82) | 45.9 | **<.001** | 32.5% (230) | 49.7% (98) | 41.2% (56) | 20.8 | **<.001** |
| The doctor downplayed my symptoms or complaints | 36.2% (389) | 39.8% (225) | 32.2% (164) | 6.6 | **.010** | 28.4% (137) | 39.8% (172) | 53.2% (75) | 32.6 | **<.001** | 31.8% (225) | 51.8% (102) | 39.7% (54) | 27.1 | **<.001** |
| I felt more like a medical case than a person with personal health needs. | 34.8% (374) | 39.6% (224) | 29.5% (150) | 12.1 | **<.001** | 24.7% (119) | 39.4% (170) | 56.7% (80) | 55.4 | **<.001** | 29.3% (207) | 50.3% (99) | 42.6% (58) | 33.8 | **<.001** |
| The doctor or other medical staff did not provide me with the necessary information | 34.4% (370) | 39.4% (223) | 28.9% (147) | 13.1 | **<.001** | 27.6% (133) | 38.2% (165) | 48.2% (68) | 24.5 | **<.001** | 30.1% (213) | 48.2% (95) | 39.0% (53) | 23.5 | **<.001** |
| I was denied diagnostic tests, even though I believed they were necessary | 28.7% (309) | 33.7% (191) | 23.2% (118) | 14.6 | **<.001** | 21.8% (105) | 32.4% (140) | 41.8% (59) | 26.0 | **<.001** | 24.9% (176) | 42.1% (83) | 32.4% (44) | 23.0 | **<.001** |
| I received an incorrect diagnosis that was later corrected by another doctor. | 28.1% (302) | 27.9% (158) | 28.3% (144) | 0.02 | .891 | 21.4% (103) | 30.3% (131) | 46.1% (65) | 34.3 | **<.001** | 23.6% (167) | 42.6% (84) | 33.8% (46) | 29.4 | **<.001** |
| I had trouble understanding the doctor's recommendations because they were not clear to me | 25.9% (278) | 27.4% (155) | 24.2% (123) | 1.4 | .229 | 23.4% (113) | 25.2% (109) | 36.9% (52) | 10.5 | **.005** | 22.2% (157) | 36.5% (72) | 32.4% (44) | 19.4 | **<.001** |
| The doctor prescribed me a drug that caused serious side effects that I had not been informed about beforehand | 20.1% (216) | 21.7% (123) | 18.3% (93) | 2.0 | .157 | 14.3% (69) | 21.8% (94) | 36.2% (51) | 33.2 | **<.001** | 16.0% (113) | 31.5% (62) | 26.5% (36) | 26.6 | **<.001** |
| The doctor spoke to me in an offensive manner | 19.3% (207) | 20.7% (117) | 17.7% (90) | 1.5 | .215 | 16.6% (80) | 19.0% (82) | 30.5% (43) | 13.6 | **.001** | 16.0% (113) | 29.9% (59) | 22.8% (31) | 20.2 | **<.001** |
| My dignity was violated by a doctor or other medical staff | 19.1% (205) | 22.6% (128) | 15.1% (77) | 9.7 | **.002** | 15.8% (76) | 18.8% (81) | 31.2% (44) | 16.9 | **<.001** | 13.6% (96) | 35.0% (69) | 25.7% (35) | 49.9 | **<.001** |
| My privacy was violated by a doctor or other medical staff | 16.8% (181) | 18.6% (105) | 14.9% (76) | 2.5 | .113 | 13.5% (65) | 19.9% (86) | 19.9% (28) | 7.6 | **.022** | 11.7% (83) | 32.0% (63) | 22.1% (30) | 47.8 | **<.001** |

Sample sizes (N) vary because some respondents selected "Other" for sex, and in questions on self-assessed health and life satisfaction, they could choose the option "This is a very sensitive question; I prefer not to answer."

**Supplementary Table 2.** Selected socio-demographic characteristics of respondents and reasons for canceling a doctor's appointment.

| **Survey items** | **Total**  **% (n)** | **Age, N=1075** | | | | | | **Satisfaction of life, N=1040** | | | | **Sex N=1075** | | | **Insurance, N= 1001** | | |
| --- | --- | --- | --- | --- | --- | --- | --- | --- | --- | --- | --- | --- | --- | --- | --- | --- | --- |
|  |  | **18–24** | **25** | **35** | **45** | **55** | **χ²(df) p** | **Very good and good** | **Very bad and bad** | **Hard to say** | **χ²(p)** | **Female** | **Male** | **χ²(p)** | **Public** | **Public and private** | **χ²(p)** |
| previous poor relations with this doctor | 38.1 (410) | 36.3 (33) | 43.4 (79) | 37.3 (81) | 41.2 (73) | 35.3 (144) | 4.5 (4) .347 | 35.8 (253) | 48.2 (95) | 37.5 (51) | 10.1 (2) **.006** | 44.7 (253) | 30.8 (157) | 21.8 (1) **<.001** | 37.9 (278) | 41.4 (111) | 1.0 (1) .316 |
| doctor's rudeness | 36.3 (390) | 40.7 (37) | 45.6 (83) | 35.0 (76) | 42.9 (76) | 28.9 (118) | 20.7 (4) **<.001** | 33.5 (237) | 48.2 (95) | 34.6 (47) | 14.6 (2)  **<.001** | 44.0 (249) | 27.7 (141) | 30.8 (1) **<.001** | 33.6 (246) | 43.7 (117) | 8.7 (1) **.003** |
| bad reviews about the doctor (e.g., on the internet) | 35.3 (380) | 45.1 (41) | 45.6 (83) | 35.9 (78) | 36.7 (65) | 27.7 (113) | 22.8 (4) **<.001** | 33.4 (236) | 44.7 (88) | 34.6 (47) | 8.6 (2) **.013** | 39.8 (225) | 30.5 (155) | 10.1 (1) **.001** | 33.4 (245) | 42.5 (114) | 7.1 (1) **.008** |
| incorrect use of the Polish language | 12.0 (129) | 14.3 (13) | 18.7 (34) | 14.7 (32) | 13.0 (23) | 6.6 (27) | 21.1 (4) **<.001** | 11.2 (79) | 18.3 (36) | 6.6 (9) | 11.6 (2) **.003** | 11.3 (64) | 12.8 (65) | 0.5 (1) .461 | 10.0 (73) | 17.5 (47) | 10.7 (1) **.001** |
| too advanced age | 11.2 (120) | 13.2 (12) | 19.8 (36) | 9.2 (20) | 10.7 (19) | 8.1 (33) | 18.8 (4) **<.001** | 9.9 (70) | 15.7 (31) | 11.8 (16) | 5.3 (2) 0.71 | 9.9 (56) | 12.6 (64) | 1.9 (1) .164 | 9.1 (67) | 16.4 (44) | 10.5 (1) **.001** |
| nationalities | 8.9 (96) | 11.0 (10) | 17.0 (31) | 12.4 (27) | 7.3 (13) | 3.7 (15) | 32.9 (4) **<.001** | 7.2 (51) | 15.7 (31) | 7.4 (10) | 14.3 (2)  **<.001** | 8.3 (47) | 9.6 (49) | 0.6 (1) .448 | 6.3 (46) | 15.3 (41) | 20.1 (1) **<.001** |
| too young | 7.1 (76) | 11.0 (10) | 11.0 (20) | 8.3 (18) | 6.8 (12) | 3.9 (16) | 13.1 (4)  **.011** | 5.9 (42) | 12.2 (24) | 5.1 (7) | 10.0 (2) .007 | 6.4 (36) | 7.9 (40) | 0.9 (1) .339 | 5.2 (38) | 10.1 (27) | 7.7 (1) **.005** |
| sex | 6.5 (70) | 15.4 (14) | 14.8 (27) | 6.0 (13) | 4.0 (7) | 2.2 (9) | 46.9 (4)  **<.001** | 5.4 (38) | 11.2 (22) | 5.1 (7) | 9.0 (2) .011 | 7.6 (43) | 5.3 (27) | 2.3 (1) .128 | 4.8 (35) | 10.1 (27) | 9.5 (1) **.002** |
| inappropriate (in my opinion) attire, makeup, or jewelry worn by the doctor | 6.1 (66) | 12.1 (11) | 12.1 (22) | 6.5 (14) | 4.5 (8) | 2.7 (11) | 26.0 (4)  **<.001** | 5.1 (36) | 12.7 (25) | 2.9 (4) | 18.1 (2)  **<.001** | 4.8 (27) | 7.7 (39) | 3.9 (1) **.049** | 3.5 (26) | 12.3 (33) | 27.2 (1) **<.001** |
| skin color | 6.1 (66) | 7.7 (7) | 12.1 (22) | 8.3 (18) | 5.1 (9) | 2.5 (10) | 23.3 (4)  **<.001** | 5.1 (36) | 10.2 (20) | 5.9 (8) | 6.9 (2) **.033** | 4.2 (24) | 8.3 (42) | 7.5 (1) **.006** | 4.0 (29) | 11.2 (30) | 18.5 (1) **<.001** |
| doctor's sexual orientation | 6.0 (64) | 11.0 (10) | 12.1 (22) | 6.5 (14) | 5.6 (10) | 2.0 (8) | 28.1 (4)  **<.001** | 4.2 (30) | 11.7 (23) | 6.6 (9) | 15.3 (2) **<.001** | 4.2 (24) | 7.9 (40) | 6.3 (1) **.012** | 3.5 (26) | 10.4 (28) | 18.3 (1) **<.001** |
